# Terrain Characterization via Machine vs. Deep Learning Using Remote Sensing

**DOI:** 10.3390/s23125505

**Published:** 2023-06-11

**Authors:** Jordan Ewing, Thomas Oommen, Jobin Thomas, Anush Kasaragod, Richard Dobson, Colin Brooks, Paramsothy Jayakumar, Michael Cole, Tulga Ersal

**Affiliations:** 1 Department of Geological Engineering, Michigan Technological University, Houghton, MI 49931, USA; toommen@mtu.edu (T.O.); jthoma2@mtu.edu (J.T.); akasarag@mtu.edu (A.K.); 2 MTRI Inc., Ann Arbor, MI 48105, USA; rdobsonmtriinc@gmail.com (R.D.); cbrooksmtriinc@gmail.com (C.B.); 3 U.S. Army DEVCOM Ground Vehicle Systems Center, Warren, MI 48092, USA; paramsothy.jayakumar.civ@army.mil (P.J.); michael.p.cole26.civ@army.mil (M.C.); 4 Department of Mechanical Engineering, University of Michigan, Ann Arbor, MI 48109, USA; tersal@umich.edu

**Keywords:** machine learning, deep learning, remote sensing, hyperspectral imaging, thermal imaging, terramechanics, mobility, terrain strength, soil

## Abstract

Terrain traversability is critical for developing Go/No-Go maps for ground vehicles, which significantly impact a mission’s success. To predict the mobility of terrain, one must understand the soil characteristics. In-situ measurements performed in the field are the current method of collecting this information, which is time-consuming, costly, and can be lethal for military operations. This paper investigates an alternative approach using thermal, multispectral, and hyperspectral remote sensing from an unmanned aerial vehicle (UAV) platform. Remotely sensed data combined with machine learning (linear, ridge, lasso, partial least squares (PLS), support vector machines (SVM), and k nearest neighbors (KNN)) and deep learning (multi-layer perceptron (MLP) and convolutional neural network (CNN)) are used to perform a comparative study to estimate the soil properties, such as the soil moisture and terrain strength, used to generate prediction maps of these terrain characteristics. This study found that deep learning outperformed machine learning. Specifically, a multi-layer perceptron performed the best for predicting the percent moisture content (R^2^/RMSE = 0.97/1.55) and the soil strength (in PSI), as measured by a cone penetrometer for the averaged 0–6” (CP06) (R^2^/RMSE = 0.95/67) and 0–12” depth (CP12) (R^2^/RMSE = 0.92/94). A Polaris MRZR vehicle was used to test the application of these prediction maps for mobility purposes, and correlations were observed between the CP06 and the rear wheel slip and the CP12 and the vehicle speed. Thus, this study demonstrates the potential of a more rapid, cost-efficient, and safer approach to predict terrain properties for mobility mapping using remote sensing data with machine and deep learning algorithms.

## 1. Introduction

Mission success for off-road military operations relies heavily on mobility (Go/No-Go) maps, which are traditionally derived from collecting in-situ measurements to quantify the soil strength of an area of interest [1,2,3,4]. Such in-situ measurements are ideally performed using a bevameter [5], but it is difficult to transport due to its large size. The use of a cone penetrometer has been the second choice due to its ease of transportation and usability [5,6]. However, how to correlate cone index values to Bekker parameters for predicting terrain parameters for physics-based vehicle mobility models is still an open research question [7]. Furthermore, although the acquisition of in-situ measurements provides accurate information on soil strength, the approach has inherent issues, such as being time-consuming and costly [8], the limitation of obtaining only point measurements [9,10], the accessibility to the area, and the risk of military deployment in potentially hazardous environments to collect the data.

To overcome these limitations of conventional methods, digital soil characterization has been identified as an alternative approach [9]. The use of remote sensing data for soil mapping has been demonstrated to be cost-effective and less time-consuming at a wide range of spatial scales compared to in-situ measurements, with reduced risk to human life [11].

Remote sensing enables the extraction of multiple soil properties to produce consistent and comprehensive soil data in space and time, facilitating soil characterization. For instance, Shahriari et al. [12] spatially predicted soil texture fractions (RMSE: sand = 15.04%, silt = 12.68%, and clay = 8.77%) in the Sistan floodplain with a hot and dry climate using a digital number of bands, band ratios, and different indices derived from Landsat 8 Operational Land Imager (OLI) data and hybrid geostatistical models. Another method for predicting the spatial patterns of the topsoil texture at a finer spatial scale (15 m) in the Attert catchment (Luxembourg, 288 km^2^) using ASTER thermal remote sensing data has been illustrated by Muller et al. [13]. Soil and crop segmentation [14] have been performed on a UAV platform with improvements in segmentation using digital surface models and an updated NDVI filter to improve the DL models.

Numerous researchers (e.g., [9,15,16]) highlighted the suitability of thermal and optical remote sensing to quantify the soil moisture content. Scheidt, Ramsey et al. [17], Lei, Bian et al. [18], Sohrabinia, Rack et al. [19], and Taktikou, Bourazanis et al. [20] observed a notable correlation between Thermal Inertia (TI)/Apparent Thermal Inertia (ATI) and soil moisture and land use/land cover types, except in densely vegetated terrains [21]. Soliman et al. [22] noted that thermal inertia is sensitive to both soil moisture and mechanical resistance. They also found that thermal inertia from a UAV versus a handheld sensor was more sensitive to in-situ moisture variability, likely due to small-scale variability on the ground. The connection between ATI and soil moisture becomes more challenging when different land cover and soil types exist [22,23]. Furthermore, estimating soil properties remotely, such as soil moisture, has limitations, such as needing ground control points for model validation [23] and normalizing predictors/indices to make them comparable between different days [24].

Optical (color) remote sensing from a UAV has been used to build 3D models for the prediction of the bearing strength of beach sand [25]. ATI has also been used with machine learning to predict soil stiffness [26]. Another study characterized lunar surface stiffness (i.e., soil strength) by examining the wheel sinkage from greyscale imagery [27]. The group could estimate the soil strength for the terrain it had already driven but lacked knowledge about the sites it had not visited yet. In other words, it still required the rover to be at the location or required on-site measurements to obtain high-resolution imagery of the wheel sinkage. All these soil parameters are critical when determining the mobility of an off-road environment.

Remote sensing-based methods gather large amounts of environmental data, which are difficult to manage by traditional methods of data analysis. With the increasing availability of remote sensing data and the development of data-driven approaches, such as machine learning (ML) and deep learning (DL), substantial progress is noted in digital soil mapping and characterization [28]. Due to their ability to handle complicated non-linear relationships among the variables, machine learning and deep learning models have been extensively utilized in soil characterization studies [28,29,30,31].

ML and DL models have also been implemented in physics-based simulations using soil slope and cone index values [2] to accurately generate mobility maps. Other studies demonstrating the capabilities of proprioceptive (e.g., wheel speed, slip, etc.) and exteroceptive (optical, LiDAR, etc.) sensors onboard have also been used for mobility prediction and classification [32,33,34,35,36], but such approaches require the deployment of vehicles in the field. Although remote sensing has been employed for mobility planning, the application provides either qualitative results [37] or mobility classification [38].

In summary, the current major approaches for generating mobility maps are either physics-based simulations, vehicle-mounted onboard sensors (on-site measurements), or remote sensing that is only qualitative or used with a mobility classification (impossible, low, medium, high). Thus, a gap is identified: efficient methods for predicting spatially distributed quantitative estimates of soil strength at fine spatial scales from remote sensing are lacking.

To address this gap, we explored the applicability of remotely sensed data from multiple sensors and ML/DL models for predicting soil strength quantitatively for mobility applications. In particular, we used thermal, multispectral, and hyperspectral data with ML (linear, ridge, lasso, partial least squares (PLS), support vector machines (SVM), and k nearest neighbors (KNN)) and DL (multi-layer perceptron (MLP) and convolutional neural networks (CNN)) models to predict the soil strength in different soil conditions and demonstrated the accuracy with field level testing using an MRZR vehicle. This approach facilitates a means for predicting the terrain properties of soil moisture and strength utilizing only remotely sensed information for off-road mobility applications, such as generating Go/No-Go mobility maps. Thus, it offers a solution to perform terrain strength predictions at larger spatial scales rapidly and efficiently, which can be helpful in assessing vehicle mobility in unstructured environments.

## 2. Materials and Methods

### 2.1. Field Sites and Soil Characteristics

**Figure 1:**
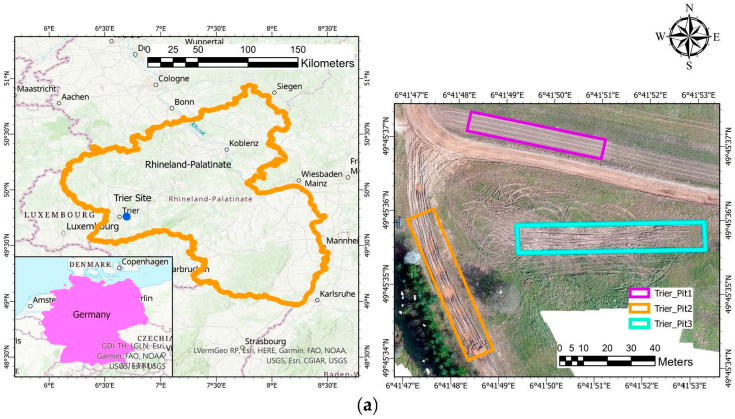

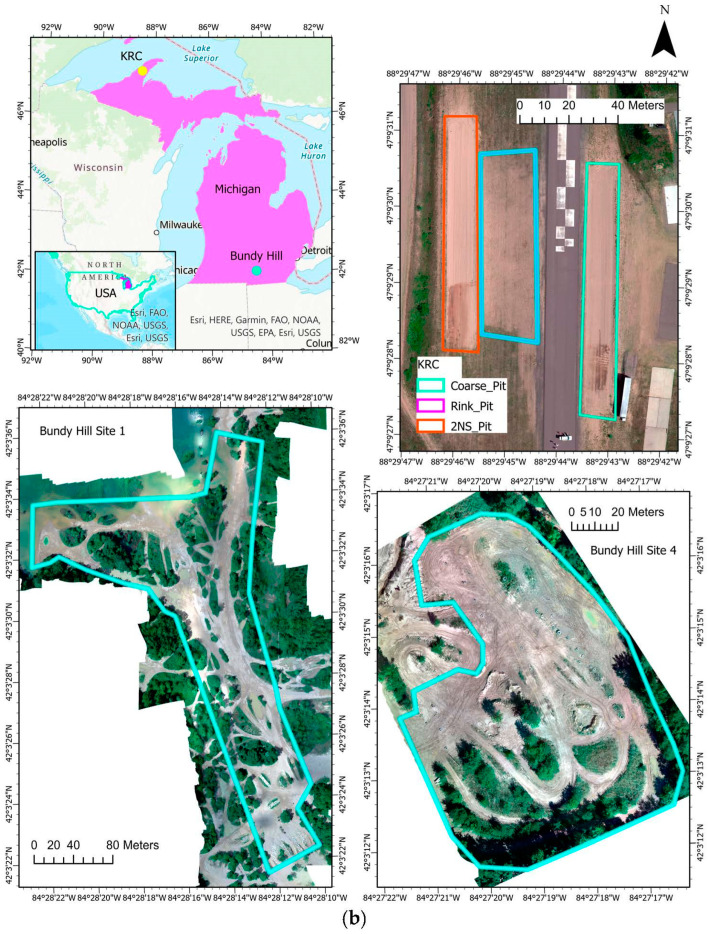
(**a**) Field site map for Trier WTD41 site, including soil pits. (**b**) Field sites in Michigan, USA (top right) KRC, (bottom left & bottom right) Bundy Hill (Sites 1 and 4, respectively).

**Figure 2:**
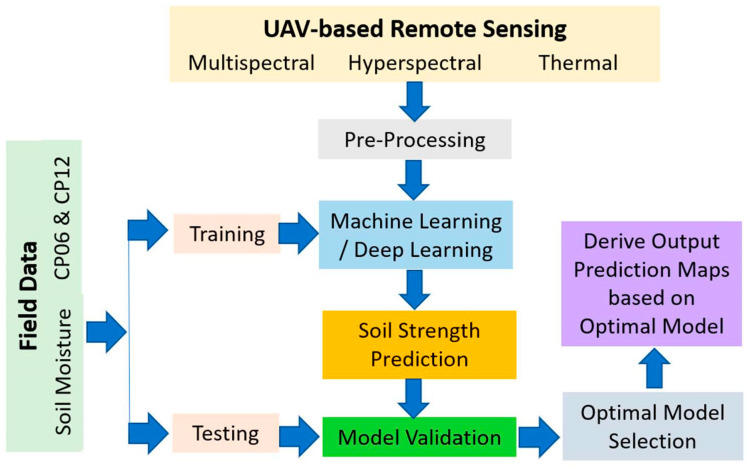
Overview of the methodological framework for this project.

Remote sensing-based soil strength characterization was performed at multiple field locations: (1) Keweenaw Research Center (KRC) (Calumet, MI, USA), Bundy Hill Off-road (Jerome, MI, USA), and WTD 41 (Trier, Rhineland-Palatinate, Germany) (Figure 1a,b). KRC and WTD 41 are mobility testing grounds that contain multiple off-road testing tracks. In addition, they conduct mobility research and perform various ground vehicle systems tests. Bundy Hill is primarily an off-road driving park but is also used for vehicle testing. Soil samples were collected from each site, and gradation tests were performed to identify the soil particle size distribution. We performed a dry sieve analysis for sandy soil and dry and wet tests (hydrometer) for the silt- and clay-dominated soils. Different soil types were present between the sites: sandy soil dominated in KRC, silty clay dominated in Trier, and Bundy Hill had a mix of sandy, silty, and clayey soils (Table 1). Figure 2 shows the methodological framework adopted in this study.

### 2.2. Remote Sensing Data Collection

Multiple sensors were used for this study to collect the data, including multispectral, hyperspectral, and thermal sensors. A Nikon D850 full-frame 45.7 mega-pixel camera was used at Trier (Germany), and a 10-band multispectral MicaSense RedEdge-MX and MX-Blue dual sensor setup were used at KRC and Bundy Hill (USA) to produce the multispectral (optical) mosaics of the area. This dual camera setup has bands that are sensitive in the 444 nm to 842 nm range with a pixel resolution of 1280 × 960 [39,40]. The hyperspectral imaging was performed using a BaySpec OCI-F push-broom sensor with a resolution of 800 pixels by the scan width and a wavelength range of 400 to 1000 nm (visible to near-infrared) [41] at all the locations. These were flown around midday to allow the sun to rise and have optimal lighting conditions and minimize any shadowing effects. Thermal imagery was collected via a radiometrically calibrated FLIR Vue Pro R [42] for Trier and a DJI Mavic 2 Enterprise Advance Thermal radiometric thermal camera [43] for KRC and Bundy Hill, both with a resolution of 640 × 512 pixels. The thermal sensors were flown in both the morning and afternoon. All the images (i.e., multispectral, hyperspectral, thermal), after mosaicking and georeferencing, were resampled to the same spatial resolution (10 cm × 10 cm) prior to the data sampling for the ML/DL modeling.

**Figure 3:**
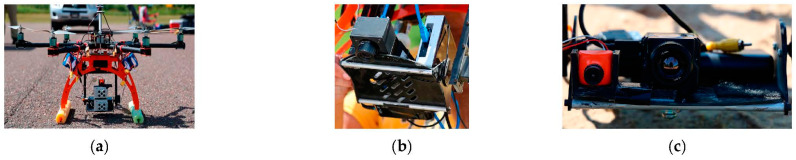
Project heavy-lift Bergen hexacopter drone mounted with various sensors. Micasense multispectral sensors (**a**), BaySpec hyperspectral sensor (**b**), and FLIR Vue Pro R thermal camera (**c**).

The data collections for all these sensors (excluding the separate DJI Mavic 2 Enterprise Advance Thermal with its built-in thermal sensor) were mounted and flown on a Bergen Hexacopter (Figure 3). Propeller Aeropoints were used to provide a 3-cm or better level positional accuracy [44] ground control points within the imagery. All the imagery (excluding imagery collected from the BaySpec OCI-F hyperspectral camera) was processed through commercially available 3D photogrammetric software. The GPS data from the Aeropoints were used during the processing to georeference and correct for geometric errors in the final products.

The collection of the multispectral imagery included steps to create radiometrically corrected data. A calibrated grey panel was imaged before and after each flight. Additionally, a Downwelling Light Sensor (DLS-2) was mounted to the top of the Bergen Hexacopter to provide additional data during collections with variable lighting conditions. This imagery was processed through Agisoft Metashape to produce a radiometrically calibrated orthoimage of each site. Multispectral imagery was used to georeference the hyperspectral imagery due to the larger field of view providing coverage of the full area and capturing more Aeropoints. Similarly, the BaySpec OCI-F hyperspectral camera required additional steps prior to the data collection to create radiometrically calibrated imagery. These steps included capturing a dark and white reference (95% white reference provided by BaySpec). The image processing was completed through BaySpec’s proprietary software.

### 2.3. In-Situ Data Collection

We collected in-situ (soil strength and moisture) data for the model calibration and validation after all the remote imagery was collected. A FieldScout Cone Penetrometer [45] was used to collect the soil strength (measuring in PSI) for the top 0–12 inches. The volumetric soil moisture content was measured using a Field Scout TDR 150 Moisture Probe [46]. Both a Trimble GeoExplorer 6000 decimeter-edition [47] and a Trimble Geo 7X [48] handheld GPS unit were used to record the exact location of the measurements using the cone penetrometer and moisture probe. Finally, soil samples were collected from each soil pit for soil characterization.

### 2.4. ML and DL Modeling

We used various ML and DL approaches to predict the soil strength and moisture using a series of predictors (i.e., multispectral, hyperspectral, and thermal images). The ML models used in this study were linear regression, ridge regression [49,50,51], lasso regression [52,53], PLS [54], SVM [2,55,56,57,58], and KNN [2]. More details of these ML algorithms are available in Ewing et al. [26] (2021).

The DL algorithms used were the MLP [59,60,61] and CNN [62,63,64], which are feed-forward neural networks. The MLP is a “fully connected” feed-forward neural network, in which all the nodes in one layer are connected to all the nodes in the next layer. The CNN functions similarly but contains spatial location information and is not “fully connected”; it is only connected to a select few of its neighboring nodes or is weighted by distance. The CNN algorithm performs sub-sampling/feature extraction of the input data before providing an output. These are both very robust models that can handle non-linear data well [29].

### 2.5. Field Validation

In addition to validating the model performance against ground truth measurements, the applicability of the predicted map for mobility planning was also tested at Site 4 at Bundy Hill using a Polaris MRZR. The MRZR was run the following day on a set loop over the area scanned the previous day. The driver was set to maintain the MRZR by going as fast as safely possible throughout the loop. This track covered both sides of the Bundy Hill Site 4 pit and had straight line portions on both sides to maintain focus on the vehicle parameters due to the terrain conditions and not from slowing down around corners. The MRZR had onboard sensors to collect proprioceptive vehicle information while driving. The Trimble handheld GPS was also used to collect the location of the MRZR throughout the course on a one-hertz basis. The data collected by the MRZR were used to derive the vehicle speed and slip ratio.

### 2.6. Predictors and Data Cleaning

#### 2.6.1. Predictor Variables

The predictors used include digital numbers of the optical (RGB) bands, thermal images (morning and afternoon), and hyperspectral imagery (75 bands) and calculated indices, such as albedo (averaged reflectance from 400–1000 nm), the soil classification index [65], the normalized difference vegetation index (NDVI), the scaled soil temperature difference ($S*∆T_{Soil}$), and the scaled ATI.

#### 2.6.2. Thermal Inertia/Apparent Thermal Inertia

The potential to absorb and store heat within a material can be measured using Thermal Inertia (TI). Thermal Inertia can be calculated [66] using the following Equation (1).

(1) $$TI=\sqrt{k\rho c},$$

Thermal conductivity (k), bulk density (ρ), and specific heat (c) still require in-situ measurements. Therefore, we turn to Apparent Thermal Inertia (ATI), which is a remote sensing approach, to approximate this TI value. ATI can be calculated using Equation (2) below [66].

(2) $$ATI=\left(1-\alpha \right)/∆T_{Soil},$$

The soil temperature change ($∆T_{Soil}$) of a material and the albedo (α) are the only variables required and can be collected remotely. The overall average reflectance from the 400–900 nm wavelength range is the albedo, which will be influenced by the surface properties (roughness, color, etc.). Different days and areas will have varying amounts of sunlight and heating based on the weather. This formula, therefore, needs to be adapted to compare different days against each other. Hence, a scaling factor (S) is needed and applied to the formula shown below in Equations (3) and (4).

(3) $$S=\frac{∆T_{Optimal\_Air}}{∆T_{Actual\_Air}},$$

(4) $$ATI_{Scaled}=\left(\frac{1-\alpha }{S*∆T_{Soil}}\right)=\left(\frac{1-\alpha }{\left(\frac{∆T_{Optimal\_Air}}{∆T_{Actual\_Air}}\right)*∆T_{Soil}}\right),$$

The optimal air temperature change ($∆T_{Optimal\_Air}$) is a constant 10 °C, which was used to ensure adequate heat change and that the soils did not become fully heat saturated. The air temperature change ($∆T_{Actual\_Air}$) is the outside air temperature difference from the morning to afternoon thermal flights.

#### 2.6.3. Data Pre-Processing

Where the in-situ measurements were taken, we assumed a 0.5 m diameter circle centered at each measurement to have uniform strength and moisture content. All the pixels within that zone in the remotely sensed imagery correspond to the measured values. All the data were pre-processed using Box-Cox (Yeo-Johnson), center, and scaling because some of the algorithms are scale invariant. The data points were divided into an 80/20 [%] (train/test) stratified random split while running the ML models. When optimizing the ML models, a K-fold cross-validation was also performed on the training data to optimize the model parameters. For DL, a stratified random 20% test/10% validation/70% training split was performed. Then, the best-performing models with optimal tuning parameters were used for testing.

Due to the high complexity of this dataset, the DL models were also run without the multispectral (RGB) predictors, to see their influence. Principal component analysis (PCA) was also performed to assess how well the model performed using a lower number of predictors. With 10 principal components, 83.6% of the overall variance was captured, which plateaued after this. Hence, 10 principal components were used for the models. This PCA analysis used all of the predictors, including the multispectral (RGB) predictors.

This approach requires multiple flights, due to the two thermal predictors, to calculate ATI. Therefore, we also examined DL models using only predictors that would require a single flight (hyperspectral and thermal only) and removed the predictors that require two temperature images (scaled temperature difference and scaled ATI). Since there were two thermal collections (AM and PM), the PM thermal data were used since they were collected directly before the in-situ measurements, which were scaled using the ratio of the actual air temperature to the thermal PM. This set of predictors was then run and analyzed using PCA. The PCA analysis showed that 10 principal components could explain 78% of the variance in the data.

## 3. Results

We predicted the soil moisture content (%) and the soil strength measured by a cone penetrometer (in PSI), averaged over the first 0–6″ and 0–12″ (CP06 and CP12, respectively). The field measurements indicated that the soil moisture and strength of the first 0–6″ (CP06) and 0–12″ (CP12) varied significantly across the sites (i.e., Trier, KRC, and Bundy Hill) (Table 2).

**Figure 4:**
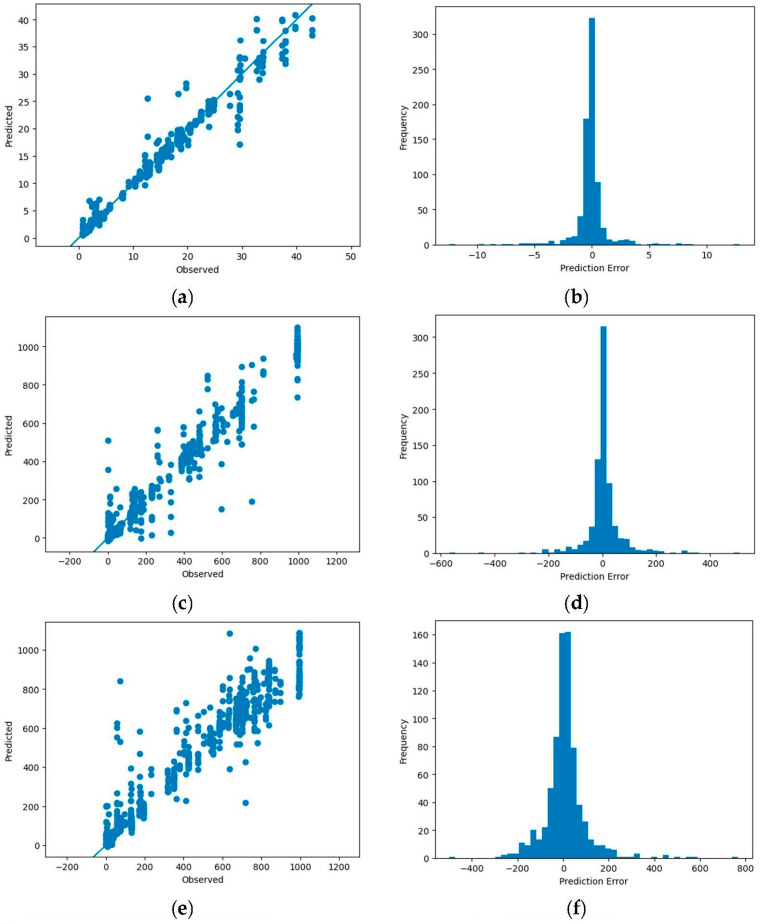
Optimal DL models using MLP (all predictors) for moisture content (**a**,**b**), CP06 (**c**,**d**), and CP12 (**e**,**f**) are shown comparing the predicted vs. true values and error histograms. Multi-picture format.

**Figure 5:**
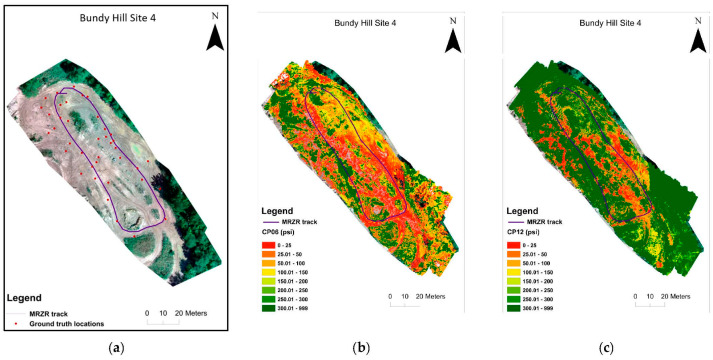
Map of Bundy Hill Site 4 showing the course track in optical mosaic with in-situ locations (**a**), the predicted CP06 map (**b**), and the predicted CP12 map (**c**).

A total of 70 in-situ measurements were taken, and the total number of samples was 3724. The model performance measures (R^2^ and RMSE) revealed that the DL models (MLP and CNN) outperformed the ML models, with the MLP performing the best overall (Table 3). The results also showed that including the RGB predictors did slightly improve the results compared to the other models. Dimensionality reduction using PCA analysis found that using 10 principal components slightly lowered the model performance, except for CP12. The PCA did perform comparably well, though using all the predictors provided the best prediction performance. While the CNN model showed good accuracy in predicting the soil moisture and strength (R^2^ > 0.85), the MLP model was the overall top model for the soil moisture and strength (R^2^ > 0.95 and had the lowest RMSE values). The single flight predictor set performed well (R^2^ > 0.87) for all the MLP models and (R^2^ > 0.84) for all the CNN models. Although these results were lower than using all of the predictors, it did show that it could provide comparable results while reducing the need for a second thermal imaging flight. The results of the best-performing models using all of the predictors are shown below in the plots for “Predicted vs. True values” and the histogram prediction error distributions in Figure 4. The optimal MLP models, built using the 0.5 m ground truth measurement buffer zones, were then applied to the full area mosaic to be used to generate prediction maps of the moisture content (%) and soil strength (CP06 and CP12) for the Bundy Hill Site 4 (Figure 5). The southwest side was stronger (yellow/green) than the northeast side (red/orange), which is visible in both the CP06 and CP12 plots. The CP12 plot emphasizes this a little clearer with the orange (0–100 PSI) northeast side compared to the darker green (250+ PSI) southwest side.

## 4. Discussion

**Figure 6:**
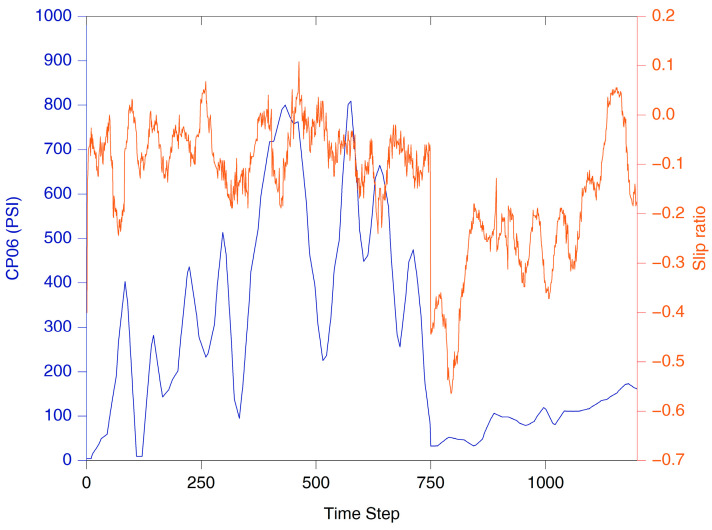
Moving Average (window size is a period of 25) plot for MRZR rear wheels showing the slip versus predicted CP06 from MLP model.

**Figure 7:**
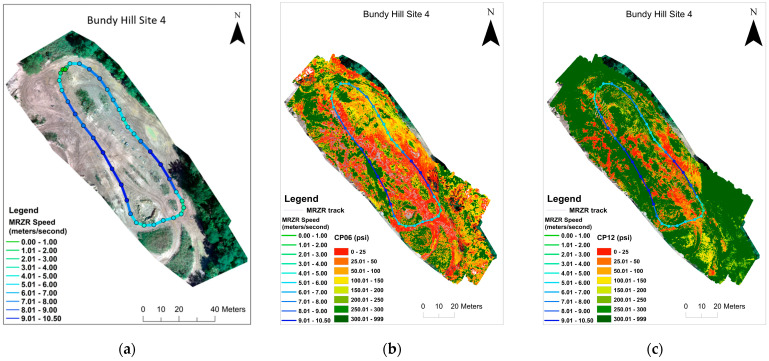
Map of Bundy Hill Site 4 showing the MRZR speed over the optical image (**a**), predicted CP06 (**b**), and CP12 (**c**) from the MLP models.

**Figure 8:**
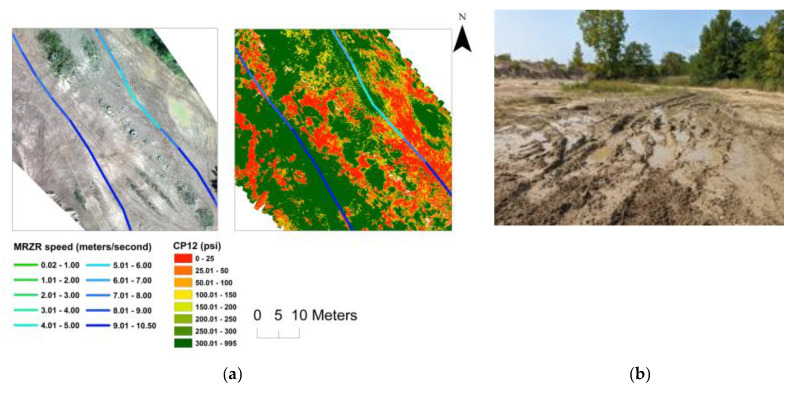
Zoomed-in image of Bundy Hill Site 4 showing the MRZR speed over the optical map and the predicted CP12 from the MLP model (**a**) and optical image of the muddy terrain (**b**).

The results demonstrate the potential of the integration of remote sensing and ML/DL models for predicting spatially distributed soil strength. The soil strength and potential for mobility depend on numerous soil characteristics [38,67], making soil strength prediction a complex non-linear problem, for which the ML and DL approaches are well suited [2,29]. We used multiple sensors including thermal, multispectral, and hyperspectral data, which adds to the higher dimensionality of this problem. As shown in Table 3, the DL models (CNN and MLP) are the best-performing models for predicting soil moisture, CP06, and CP12. Better performance of the DL models was expected as the DL models perform well with larger datasets and high dimensionality [31]. It was also demonstrated that the capability of using a “single flight” set of predictors to do these predictions is a viable option as well as PCA for helping to reduce the dimensionality. The utility of the predicted soil strength map for mobility planning was tested using an MRZR vehicle at Bundy Hill Site 4. A comparison of the soil strength, CP06, along the MRZR path and the slip ratio of the rear wheels during the timeframe (in seconds) it took to run through the course indicates a general agreement with an inverse relationship (Figure 6). However, deviations from the generalized behavior were also noted, which could be attributed to the uncertainty of the soil strength predictions, errors from MRZR sensors, and localized soil variations. The variability in the soil strength was also reflected in the speed of the MRZR throughout the course, where the MRZR moved slower in regions with weaker soil and vice versa, even with the driver trying to maintain a fast, even speed. In addition, slower speeds could also have been due to the muddy portions of the track causing higher slip and, therefore, slower movement (Figure 7 and Figure 8). This is a limited dataset, and more testing is needed for quantitative validation. Nevertheless, the results point to an important future research direction.

One of the limitations of this study is the lack of soil textural resolution, where KRC has only a single soil type per pit (mostly sandy) and Trier has only one soil type (silty clay) in different conditions and varying levels of organic content. Bundy Hill is the only location that is characterized by naturally deposited/mixed soils (Table 1). However, validation of the approach in different soil types and different moisture regimes is needed as part of future research.

Another factor affecting the model performance is the drastic variation in the soil strength at a local scale, e.g., due to the presence of rock fragments based on our observation of the field conditions. With the wide variety of possible soil types and conditions, implying a diverse and larger training dataset, the model is expected to be updated to yield more accurate prediction maps.

Finally, in this study, we used thermal emissivity from the soils in the morning and afternoon, which limits this approach to an offline application. An approach to make the soil strength predictions available online is highly desirable and is, thus, identified as another important future research direction.

## 5. Conclusions

In conclusion, this study explored the use of remotely sensed data to predict useful terrain characteristics, such as soil strength and soil moisture, using various sensors and ML/DL methods. It utilized hyperspectral, multispectral, and thermal imagery to build prediction models for the soil moisture content, CP06, and CP12. Overall, the DL algorithms outperformed the ML ones, with the MLP predicting the best overall for the soil moisture (R^2^ = 0.967/RMSE = 1.55), CP06 (R^2^ = 0.953/RMSE = 67), and CP12 (R^2^ = 0.917/RMSE = 94). These models also showed usefulness for an MRZR when determining vehicle mobility. It is acknowledged that this is a preliminary observation, as it was performed at only one location with the final models, and more work is needed to quantify these relationships better and over a wider variety of soil types.

Overall, this methodology is a much quicker, more cost-effective, and safer approach to characterize the terrain properties versus the current approach of sending soldiers into unknown terrain to perform in-situ measurements, which can take more time to collect and put the soldiers’ lives at risk. The proposed method also generates more detailed maps over larger areas rather than using a few point measurements to generalize an area.

Future work will aim to better generalize the proposed models by adding more soil types with various moisture conditions. It is desirable to investigate the influence of organic content being present, which would also influence the soil strength and moisture content and play a role in shading. Scaling the proposed solution from a UAV-mounted one to satellite platforms is also important to see the potential for the scalability of the model and test the applicability of other remote sensing indices on these satellite platforms to help improve the predictions and calculate them over larger areas. A larger database of more soil types and field sites would allow one to explore more machine/deep learning algorithms and possible multi-level approaches to the models to pursue better predictive capabilities. Lastly, investigating how to convert this single-flight approach into a real-time implementation for instantaneous predictions in the field is also of interest.

## Figures and Tables

**Table 1:** Soil characteristics at locations used in this study.

#	Site	Pit	%Gravel	%Sand	%Fine
1	KRC	2NS	3.3	95.6	1.1
2	KRC	2NS	1.7	97.4	0.9
3	KRC	Rink	7.2	86.6	6.2
4	KRC	Rink	4.7	90.4	4.9
5	KRC	Coarse	3.0	95.7	1.3
6	KRC	Coarse	1.8	97.3	0.9
7	Bundy Hill	S1	0.4	25.4	74.2
8	Bundy Hill	S1	0.0	48.3	51.7
9	Bundy Hill	S1	2.0	92.3	5.7
10	Bundy Hill	S1	6.9	79.3	13.8
11	Bundy Hill	S1	3.0	94.7	2.3
12	Bundy Hill	S4	2.6	26.5	70.9
13	Bundy Hill	S4	8.9	38.4	52.7
14	Bundy Hill	S4	19.1	44.7	36.2
15	Bundy Hill	S4	1.3	68.8	29.9
16	Trier	Pit 1	6.9	38.2	54.9
17	Trier	Pit 1	4.0	48.4	47.6
18	Trier	Pit 1	7.2	43.2	49.6
19	Trier	Pit 2	7.4	40.8	51.8
20	Trier	Pit 2	6.2	41.1	52.7
21	Trier	Pit 3	8.1	36.9	55.0
22	Trier	Pit 3	4.9	42.6	52.5

**Table 2:** Table of the different response variables: moisture content (%), averaged cone penetrometer (PSI) for the first 0–6″ (CP06) and 0–12″ (CP12).

Location (Pit)	Moisture Range	Moisture Mean	CP12 Range	CP12 Mean	CP06 Range	CP06 Mean
Trier (Pit1)	10.3–14.7	12.3	37–195	109	11–116	46
Trier (Pit2)	14.7–24.8	19.9	58–995	436	0–995	264
Trier (Pit3)	14.7–16.4	15.8	7–837	512	13–701	294
KRC (Coarse)	1.0–2.9	1.9	0–702	148	0–451	71
KRC (Rink)	1.1–2.1	1.6	175–995	489	0–995	251
KRC (2NS)	0.8–4.6	2.3	0–812	112	0–655	66
BH (S1)	4.9–27.8	14.2	77–821	640	44–671	416
BH (S4)	0.8–42.9	19.2	73–995	740	15–995	586

**Table 3:** Table of prediction accuracy (R^2^) and Root Mean Squared Error (RMSE) for all the regression models used in this study and the corresponding response variables.

R^2^/RMSE for Each Regression Model			
Algorithms	Moisture Content (%)	CP06 (PSI)	CP12 (PSI)
Linear	0.508/6.00	0.478/231	0.524/229
Ridge	0.510/5.99	0.483/230	0.527/229
Lasso	0.508/6.01	0.489/229	0.531/228
PLS	0.044/8.37	0.140/297	0.109/314
SVM	0.351/6.90	0.197/287	0.539/226
KNN	0.614/5.32	0.662/186	0.647/197
MLP (All predictors)	0.967/1.55	0.953/67	0.917/94
CNN_(All predictors)	0.895/2.78	0.895/100	0.852/126
MLP (No RGB)	0.951/1.89	0.934/79	0.940/80
CNN_(No RGB)	0.860/3.21	0.906/95	0.867/119
MLP_(PCA 10)	0.929/2.21	0.932/86	0.944/80
CNN_(PCA 10)	0.913/2.44	0.918/94	0.902/106
MLP (Single flight predictors)	0.932/2.23	0.906/95	0.924/90
CNN (Single flight predictors)	0.862/3.18	0.866/113	0.846/128
MLP (Single flight predictors with PCA 10)	0.887/3.01	0.877/110	0.903/104
CNN (Single flight predictors with PCA 10)	0.874/3.18	0.863/116	0.858/126

## Data Availability

The data are not publicly available due to proprietary restrictions and confidentiality agreements with third-party organizations involved in the collection and analysis of the data.

## Distribution Statement

DISTRIBUTION A. Approved for public release; distribution unlimited. OPSEC # 7377.
